# Systemic Immunomodulatory Therapy, Anterior Chamber Inflammation, and the Use of Topical Corticosteroids in Juvenile Idiopathic Arthritis-Associated Uveitis: A Long-Term Real-Life Observational Study

**DOI:** 10.3390/jcm15020812

**Published:** 2026-01-19

**Authors:** Marija Barišić Kutija, Sanja Perić, Mario Šestan, Petra Kristina Ivkić, Martina Galiot Delić, Tomislav Jukić, Josip Knežević, Marijan Frković, Vladimir Trkulja, Marija Jelušić, Nenad Vukojević

**Affiliations:** 1 Department of Ophthalmology, University Hospital Centre Zagreb, 10 000 Zagreb, Croatia; marija.barisic@gmail.com (M.B.K.);; 2 Department of Ophthalmology, University Hospital Centre Zagreb, University of Zagreb School of Medicine, 10 000 Zagreb, Croatianvukojev@gmail.com (N.V.); 3 Department of Pediatrics, University Hospital Centre Zagreb, University of Zagreb School of Medicine, 10 000 Zagreb, Croatia; 4 Department of Pharmacology, University of Zagreb School of Medicine, 10 000 Zagreb, Croatia

**Keywords:** juvenile idiopathic arthritis, uveitis, topical corticosteroid, anterior chamber inflammation, biological treatment, conventional immunosuppressive treatment, anterior chamber inflammation, outcome measures

## Abstract

**Background:** Juvenile idiopathic arthritis-associated uveitis (JIA-U) is a rare condition, and assessment of the efficacy of disease-modifying antirheumatic drugs, synthetic (sDMARD) or biological (bDMARD), in randomized trials is hindered by this fact. **Methods:** In this prospective longitudinal study, we observed 38 children aged 1.3 to 15.2 years, with 69 eyes affected with JIA-U for 1970 overall eye examinations (6–59, median 16) irregularly scattered across 4.4–87.6 months (median 21.6) of follow-up, with on- and off-periods of DMARD use and use of topical treatments. **Results:** With adjustment for several time-invariant and time-varying covariates, periods of exposure to sDMARD vs. no DMARD exposure were associated with peak benefits of 15–20% lower probability of having more severe anterior chamber (AC) inflammation and a similar relative reduction in the daily use of topical corticosteroids (TCS). Periods of bDMARD exposure or of bDMARD + sDMARD exposure vs. no DMARD use were associated with peak benefits of an around 50% reduction in the probability of having more severe AC inflammation, and peak benefits of an around 60–65% reduction in TCS use. **Conclusions:** The observations regarding bDMARD (only) or bDMARD + sDMARD exposure are in agreement with the extent of benefits suggested for adalimumab vs. placebo (+background sDMARD) in the only existing randomized trial in this setting evaluating AC inflammation and TCS use.

## 1. Introduction

Juvenile idiopathic arthritis-associated uveitis (JIA-U) is the most common extra-articular manifestation of JIA, and the main cause of childhood uveitis in developed countries [1,2,3,4,5,6,7]. Despite advances in JIA-U management [8,9], it remains one of the leading causes of vision impairment in childhood [10,11,12,13,14,15,16], and markedly reduces quality of life [17].

The goal in long-term therapy for chronic, refractory, or recurrent JIA-U is to suppress inflammation in the anterior chamber (AC) and to minimize the use of systemic and topical corticosteroids (TCS) [12,18,19] in order to avoid systemic and local adverse effects. Immunomodulatory treatment (IMT), which includes conventional (synthetic) and biologic disease-modifying antirheumatic drugs (sDMARD, bDMARD, respectively), is a corticosteroid-sparing therapy that may improve the disease course and outcomes [2,8,10,20,21,22].

Assessing treatment efficacy through randomized placebo-controlled or comparative trials (RCTs) in rare diseases is hindered by the low incidence/prevalence of eligible patients, a well-known issue in JIA-U [23,24]. In particular, the eligibility of JIA-U patients in whom IMT is indicated is even lower [14,25,26,27,28,29]. Only one moderately sized trial has assessed a bDMARD (adalimumab) (adalimumab versus placebo on top of a stable methotrexate (MTX)) with indicators of AC inflammation and systemic or topical corticosteroid use among primary and secondary endpoints [22]. Observational (real-life) data complement the estimates generated in RCTs by illustrating the extent to which treatment potentials are transferred to daily life (effectiveness), since they could be modified by circumstances not captured or intentionally avoided in RCTs. In rare diseases like JIA-U, observational data are particularly important, not only due to the lack of estimates from randomized settings, but also due to the complexity of the condition and a variety of intertwined factors that may affect the disease course and response to treatment [2,22,30]. Efforts towards standardized outcome measures and reporting across clinical studies evaluating treatment efficacy in JIA-U should increase the relevant knowledge base to define recommendable choices [31,32,33]. However, existing observational data typically stem from methodologically variable studies with different definitions of the outcomes and outcome measures, and with heterogeneous reporting [8,20,21,31,34,35,36,37,38,39]. As an illustration, we used two recent systematic reviews of the literature [34,35] to extract the main methodological characteristics of several larger (>20 patients) observational studies assessing the efficacy of IMT in pediatric JIA-U [38,40,41,42,43,44] (Appendix A). With respect to IMT specifically, this has two major consequences: (i) comparison of the results across studies is difficult or impossible; (ii) although “qualitative” conclusions are closely similar (IMT is or may be beneficial in JIA-U), it is difficult or impossible to generate reasonably accurate estimates of this benefit, including the type and extent of benefit that one could expect in daily treatment of JIA-U by introducing compounds from the IMT armamentarium.

In this context, we aimed to estimate the benefits (effectiveness) of systemic IMT, conventional or biological, in pediatric JIA-U patients over a long(er) follow-up period. Although several outcomes are informative about various aspects of the disease [32,33], we focused on two core ophthalmological outcomes: AC inflammation graded by the Standardization of Uveitis Nomenclature (SUN) criteria [45,46,47], and the extent of TCS use.

## 2. Materials and Methods

This longitudinal study was conducted at the Department of Ophthalmology, University Hospital Centre Zagreb, University of Zagreb School of Medicine, Croatia, between January 2011 and January 2018. The study enrollment period was up to 1 June 2017, in order to allow for at least a 6-month follow-up for the last enrolled patient. The study was strictly observational, in that study-related procedures pertained exclusively to the structured collection of the data of interest—no diagnostic or therapeutic procedure, nor the pattern of follow-up visits related to either JIA or JIA-U, were affected by study participation, and they remained solely at the discretion of the attending consultant. All ophthalmological examinations were conducted at a single site by the same consultant.

### 2.1. Patients and Patient Management

Those eligible for inclusion were (i) children aged 1 to 18 years, (ii) with verified JIA-U receiving sDMARD and/or bDMARD [48], and (iii) had written informed consent given by parents/guardians for the use of anonymized data for research and scientific publication. The starting point of observation was the first visit to our Department with verified JIA-U during the index period, whether incident (first diagnosis of JIA-U) or prevalent (e.g., the first control visit of patients with ongoing JIA-U during the index period, or prevalent patients referred from other institutions). The follow-up period lasted until 1 January 2018, or until loss to follow-up for any reason resulting in discontinuation of management at our institution. In such a case, the last visit recorded at our institution was the end of follow-up. Patients with uveitis of other etiologies and those with intense corneal opacities, disabling assessment of the cell counts in AC, were not included. Post hoc, we excluded a few initially enrolled patients with follow-up shorter than 3 months, as we considered this period insufficient for reliable evaluation and likely to introduce bias (e.g., a chance that findings would not be representative of disease evolution or response to treatment).

Patients were managed by a team of pediatric ophthalmologists and rheumatologists. Topical treatments (TCS drops or ointment, topical nonsteroidal anti-inflammatory drugs (NSAID), mydriatics) were steered by ophthalmologists, whereas systemic treatments (corticosteroids, NSAID, sDMARD, or bDMARD) were coordinated between specialists, and could be indicated based on the JIA status or JIA-U status, in line with the professional guidelines [2,8,9]. The first-line sDMARD was MTX, whereas leflunomide or mycophenolic acid were used sporadically as second-line options. The first-line bDMARDs were anti-TNF-alpha compounds, predominantly adalimumab, occasionally infliximab or etanercept. When adalimumab treatment was considered ineffective, the next-line choice was tocilizumab, or occasionally, etanercept/infliximab. If judged ineffective, first-line etanercept/infliximab was replaced with adalimumab, followed by tocilizumab if required. Similarly, treatments could be transiently (e.g., infection, side effects) or permanently withdrawn as deemed appropriate by the attending consultant. The pattern of scheduled ophthalmological follow-up visits was at the discretion of the attending consultant, depending on current AC inflammation severity. However, compliance with scheduled visits and treatment compliance (topical, systemic) could not be influenced beyond straightforward instructions to patients emphasizing their importance. Consequently, intervals between actual visits ranged from a few days or weeks to several months or even longer, influenced by unpredictable disease developments or intercurrent events (e.g., intensification, adverse events, local infections), and by patient-related factors. Appendix B. illustrates patterns of actual ophthalmological visits, findings, and treatments in two of the included patients. Overall, actual patient management reflected routine practice. Since all available data were considered without predefined study schedules in a controlled study protocol, the dataset is characterized by variability in examination frequency, follow-up time, demographic characteristics, and patterns of IMT.

### 2.2. Exposures, Outcomes, and Covariates of Interest

Exposures of interest were conventional and biological IMTs, or their combination: all sDMARDs were considered jointly (with a predominance of MTX), and all bDMARDs were considered jointly (with a predominance of adalimumab), as were their combinations. Exposure to these compounds was considered as a time-varying treatment with each patient contributing both “on-” and “off-” periods.

Outcomes of interest were the intensity of AC inflammation, graded as 0, 0.5, 1, 2, 3, or 4 (SUN criteria [46,47]), and the average total daily number (over the preceding week) of TSC applications (solution and ointment combined). Patient age at the start of follow-up, sex, age at JIA and at JIA-U onset, and whether the JIA was oligoarticular (vs. all other types) were recorded at the initial visit. Along with exposures and outcomes, other variables recorded at each visit were those considered possibly related to either exposures or outcomes, or both, directly or through mutual interplay: other systemic treatments (corticosteroids, NSAIDs), local findings (glaucoma or status post glaucoma, synechiae, cataract or status post cataract, keratitis) and local treatments (topical NSAID, mydriatics).

### 2.3. Data Analysis

The present data are specific, in that (i) patients underwent unequal numbers of unequally spaced follow-up visits, and their timing was likely driven by factors related and unrelated to the treated condition; (ii) indications for the installment of IMTs varied over time, and could have been determined not only by the ophthalmological status, but also by the underlying disease (JIA), and could have been commenced even when the local ophthalmological status per se would not have required them. As a consequence, individual patients contributed both “on-treatment” and “off-treatment” data, where the former occasionally might not have been informative about the treatment effect since the ophthalmological status was stable. Likewise, intercurrent events (e.g., infections) might have postponed or interrupted the treatment that was actually needed, resulting in enhanced AC inflammation or TCS use, thus “inflating” the difference between “on” and “off” treatment periods; (iii) in a setting with a varying treated condition (JIA-U), with a varying indicator (AC inflammation) prompting TCS or DMARD use, the basic condition of the temporal sequence in which presumed cause (treatment) precedes the outcome (e.g., local inflammation) is expectedly violated over time—the fact that a treatment was present at a certain time point could have been the outcome, and disease severity could have been a cause. In a classical observational cohort study with a clearly defined baseline and equally spaced post-baseline visits, time-varying treatment (arising from, e.g., inadequate compliance or intercurrent events) can be modelled by inverse probability of treatment weighting in marginal structural models, g-estimation of the structural nested models, or by g-computation [49], but none of these options were suitable for the current data structure. With this in mind, we aimed to estimate associations between exposures and outcomes of interest, while accounting for a reasonable number of covariates selected based on medical/biological rationale (“independent associations”). For interpretation of these associations, we relied on subject matter knowledge, and not exclusively on the formal proofs of no-reverse-causation or lack of selection.

If, repeatedly over the observational period, exposure to, for example, bDMARDs is associated with both less extensive TCS use and milder AC inflammation, while controlling for concomitant use of possibly interfering systemic treatments (corticosteroids, NSAIDs, sDMARD), topical treatments (topical NSAID, mydriatics), or local conditions that may influence decisions about topical or systemic treatment use (e.g., keratitis, cataract, glaucoma, synechiae), the association is suggestive of a causal effect. This inference is strengthened if effect estimates remain reasonably insensitive to residual confounding.

For the analysis of total daily TCS applications and the SUN score of the AC inflammation, the unit of observation was the eye. We fitted generalized mixed models for unequally spaced longitudinal data, with time in months as a continuous variable, and eye nested in patient as a random effect to account for correlation between repeated measures in the same eye and subject, and correlation between eyes in the same subject.

The total number of daily TCS applications/24 h was analyzed by fitting a Poisson model with fixed effects: (i) time-invariant covariates—age at the start of observation, age at JIA-U onset, and whether JIA was oligoarticular; (ii) time-varying covariates—use of systemic corticosteroids, use of systemic NSAIDs, presence of keratitis, synechiae, cataract or status post-cataract, glaucoma or status post-glaucoma, use of mydriatics, use of topical NSAID, and the SUN score of AC inflammation; (iii) time (in months) modelled as restricted cubic spline with 5 knots (placed by the Harrell’s rule); (iv) time-varying treatments of interest—sDMARD, bDMARD. A three-way interaction between time, sDMARD, and bDMARD was used to estimate differences between (a) bDMARD use vs. no DMARD, (b) sDMARD use vs. no DMARD, and (c) combined bDMARD and sDMARD use vs. no DMARD at different times since the start of observations (3, 6, 12, 18, 24, 30, 36, 42, and 48 months). The effect measured was the rate ratio, i.e., the relative (%) difference in TCS use.

The SUN score of AC inflammation was modelled as an ordered categorical variable (cumulative logit model). Since a score of 2 or 3 was observed only in a small fraction of the 1970 individual eye assessments, it was collapsed to 3 levels (0, 0.5, and 1 or higher). The fixed effects were the same as in the analysis of TCS use, except that (i) time was modelled as linear, since for cumulative logit models, cross-product matrix cannot be obtained for constructed effects (restricted cubic spline for time); (ii) while AC inflammation was a covariate in the analysis of TCS applications, TCS use was not included as a covariate in the analysis of the association between DMARD use (exposure) and AC inflammation (outcome). We reasoned as follows: (a) a certain level of AC inflammation prompts TCS use/intensification, and may also prompt the use of DMARDs. The expected benefit of introducing DMARDs (treatment) is to reduce the TCS use (outcome) for a given AC inflammation (a condition requiring either or both treatments); (b) in the RCT of adalimumab vs. placebo [22], the primary composite endpoint implied that for the avoidance of “treatment failure,” at least some improvement was required in the AC inflammation, indicating a benefit of adalimumab on this disease characteristic. At the same time, however, the use of TCS was restricted [22]. Current data were generated in daily practice without restrictions on TCS use, and TCS use was likely commonly prompted by the outcome of interest (reverse causation)—we deemed that conditioning on TCS use while assessing the association between exposure to DMARDs and AC inflammation was more likely to induce than to reduce bias. The outcome measured was the odds ratio, indicating the relative odds of having more severe AC inflammation. For data presentation, odds ratios were converted to relative risks by the optimal approximate conversion [50]: cumulative probabilities of having SUN score of 0.5 or 1+ were high (>10% of examined eyes); hence, odds ratios were numerically different from relative difference between treated and untreated eyes in probabilities (risks). Although the relative risk for such conversion is still somewhat biased vs. the true risk, it is less biased than the odds ratio and is more intuitive for interpretation. Finally, since AC inflammation and TCS use are closely correlated, the two were analyzed jointly by fitting a generalized model for joint analysis of binary (AC inflammation dichotomized to score 0 vs. any score > 0) (logit, with odds ratio finally converted to relative risk) and count data (number of TCS daily applications) (Poisson, rate ratio), with time modelled as a restricted cubic spline, and with fixed effects as in other models. All models employed robust (sandwich) variance estimation and Laplace approximation. All calculations were performed in SAS for Windows version 9.4 (SAS Inc., Cary, NC, USA).

To assess the sensitivity of the generated estimates to residual confounding, we calculated the E-value. This is expressed on a relative risk scale and quantifies the strength of association between (hypothesized) residual confounder(s) and both the exposure and the outcome that is needed to explain away the observed estimate [51].

## 3. Results

### 3.1. Eligible Patients and Follow-Up Data

We observed 38 children [32 (84.2%) girls] aged 1.3–15.2 years at the start of the observation, most [21 (55.2%)] suffering from oligoarticular JIA (Table 1). In 16 (42.1%) children, JIA-U was incident without complications, in an additional 11 (28.9%), it was incident with complications, and 11 (28.9%) children were referred to our institution with JIA-U previously treated elsewhere. Half of the children suffered JIA-U complications at the start of the observation (Table 1). Systemic treatment with sDMARDs, almost exclusively MTX, was already in place or was started at the first visit for 24 (63.1%) children, systemic corticosteroids were in place for 4 (10.5%), and NSAIDs for 18 (47.4%) children. At the first visit, a bDMARD was started or in use for 8 (21.0%) children (Table 1).

Of the 38 enrolled children, JIA-U was bilateral in 31 and unilateral in 7 children, for a total of 69 eyes. At the starting visit, most eyes had a SUN score of 0.5 or 1 [20 (29.0%) and 26 (37.7%), respectively] (Table 1), 9 (13.0%) had a score of 0, and no eye was scored 4+ (Table 1). The most common complications were cataract in 17 (26.4%) eyes, synechiae in 20 (29.0%) eyes, and keratopathy in 15 (21.7%) eyes (Table 1). At the start of the observation, all 38 children were administering TCS as drops or ointment to at least one affected eye, and 33 were using both. Of the 69 eyes, 8 were untreated with TCS, 61 were treated with TCS drops, and 52 with TCS ointment (combined with drops) (Table 1). The total number of daily TCS applications ranged from 0 to 20, with a median of 6 (Table 1).

Patients were followed-up with a median of 21.6 months (range 4.4 to 87.6, quartiles 7.6 and 40.2 months). During this time, they were examined on 6 to 59 occasions (including the initial visit, with a median of 16, and quartiles of 8 and 27). The time between visits varied from a few days to several months. Overall, 1970 eye examinations were performed.

Table 2 summarizes data considered in the present analysis grouped in 6-month intervals. Within the initial 6-month period, all 38 were examined at least once, and for a maximum of 12 times (median 6.5), resulting in 476 examined eyes. The number of examined children and eyes gradually declined over time. Between months 48 and 54, fewer than 20 children were examined, and this number progressively declined further (Table 2). Hence, we used all available data across time in analytical models, but we generated estimates of interest only up to month 48.

Across the time intervals, most eyes were treated with TCS at the time of examination, with sDMARD (MTX) in use. Approximately 30% of all eye examinations were undertaken while bDMARDs were in use (Table 2).

### 3.2. sDMARDs, bDMARDs, and Their Combination vs. AC Inflammation and TCS Use

**Figure A1:**
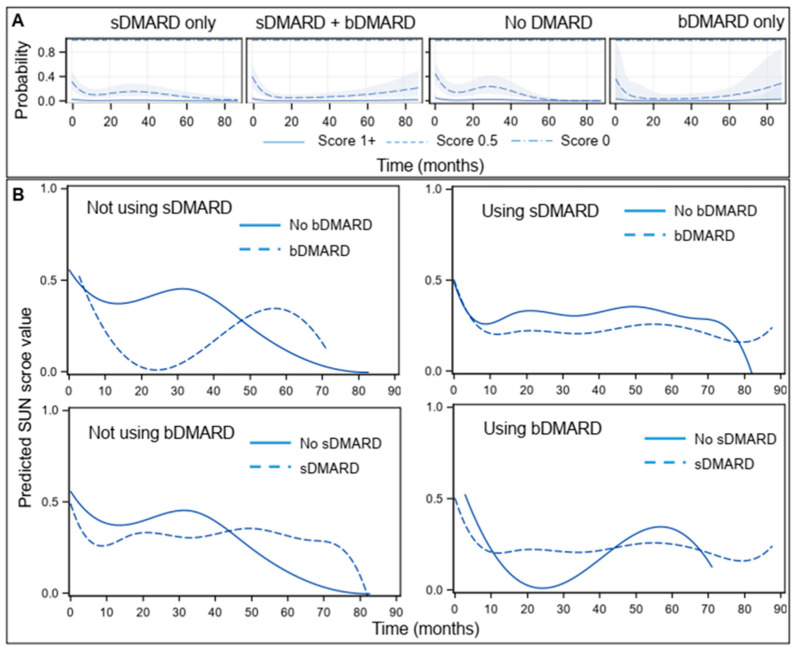
(**A**). Adjusted predicted probabilities of AC SUN score values 0, 0.5, and 1+ over time for eyes assessed while sDMARDs, bDMARDs, both, or none were in use. (**B**). Predicted AC SUN score values with bDMARD vs. no bDMARD use, while using or not using sDMARD, and vice versa.

**Figure A2:**
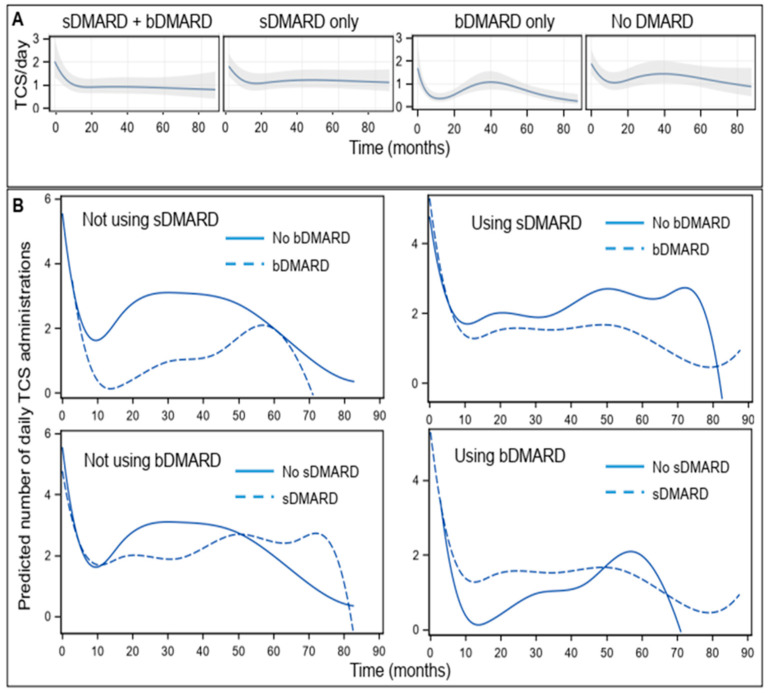
(**A**). Adjusted predicted daily TCS applications over time for eyes assessed while sDMARDs, bDMARDs, both, or none were in use. (**B**). Predicted number of daily TCS applications with bDMARD use vs. no bDMARD use, while using or not using sDMARD, and vice versa.

Table 3 summarizes adjusted predicted probabilities of SUN score and the predicted average number of daily TCS applications at specific time points generated in separate models for the AC inflammation (AC SUN score) (cumulative logit) and TCS applications (Poisson). Appendix C, Figure A1 illustrates data for the SUN score categories, and Figure A2 illustrates data for the TCS applications over the entire observed period.

The probabilities of SUN scores of 1+ or 0.5 gradually declined over time under all conditions of interest: no DMARD in use, sDMARD in use, bDMARD in use, or both sDMARD and bDMARD in use (Table 3). Across most time points, the probability of a score of 0 was the highest, and the probability of a score of 1+ or 0.5 was the lowest for eyes assessed when bDMARD (only) was in use. The values were similar for assessments when both sDMARD and bDMARD were in use (Table 3). The probabilities for assessments while sDMARD was in use appeared somewhat more favourable than those obtained with no DMARD in use, but not unequivocally (Table 3). The total number of daily TCS applications oscillated over time under all four conditions, but the values appeared consistently lower for the condition of bDMARD use or both sDMARD and bDMARD use, compared to no DMARD or sDMARD use (Table 3).

**Figure 1:**
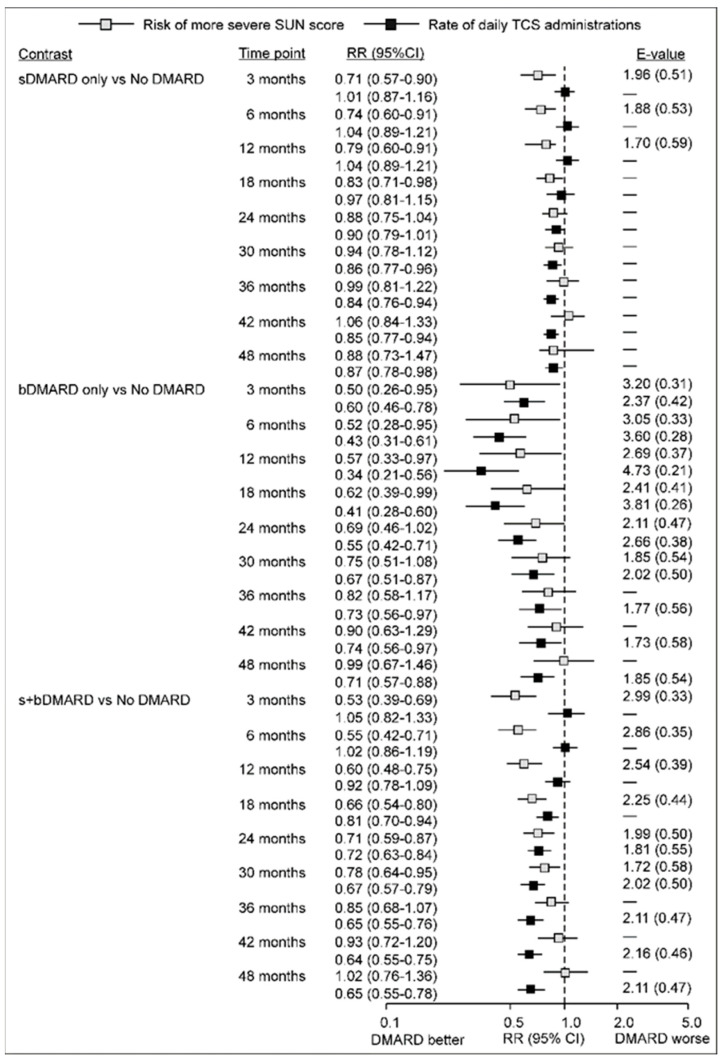
Adjusted association between exposure to DMARDs and the probability of having a more severe SUN score (0.5 vs. 0, or 1 vs. 0.5 vs. 0), and the number of daily topical corticosteroid (TCS) applications: differences between eyes assessed when sDMARD only, bDMARD only, or both s+bDMARDs were in use, and eyes assessed when no DMARDs were in use. The two outcomes were analyzed in separate models. Estimates are relative risks and rate ratios (RR), respectively. E-values were calculated only for point-estimate RRs < 0.80 with upper CI limit < 1.0 or slightly >1.0, as a strength of association of residual confounder(s) needed to “push” the observed point-estimate to RR = 0.90 (considered a likely irrelevant difference). Estimates ≥ 0.80 (regardless of upper CI limit) were close to 0.90 anyway. The first number indicates association with the exposure (fold difference in prevalence of the confounder among the exposed compared to controls), and the bracketed number (its inverse) is the strength of the confounder effect (as RR). (s,b)DMARD—synthetic/biological disease-modifying antirheumatic drugs; SUN—standard uveitis nomenclature; TCS—topical corticosteroids.

Figure 1 summarizes the estimated differences at specific time points after the start of observation in the probability of a more severe SUN score and in the total daily number of TCS applications between eyes assessed when an sDMARD (only) or a bDMARD (only) or both s+bDMARD were in use vs. eyes assessed when no DMARD was in use with each outcome analyzed in a separate model.

Regarding specific contrasts (Figure 1), (i) between months 3 and 18, the risk of a more severe SUN score appeared 20–30% lower under exposure to sDMARD compared to no DMARD, while the rate of daily TCS applications appeared similar (RRs around 1.0). After that, the risk of more severe SUN scores tended to be similar under sDMARD and no DMARD, but the rate of TCS applications appeared 10–15% lower under the former condition; (ii) a similar pattern was observed for eyes assessed under s+bDMARD exposure compared to no DMARD, except that the difference in risk of more severe SUN scores was more pronounced and persistent: it varied from being 50% lower at 3 months to 22% lower at month 30 of the observation, whereas between month 18 and month 48 of the observation, s+bDMARD exposure was associated with a 20% to 35% lower rate of TCS use; (iii) estimates generated for the bDMARD vs. no DMARD contrast had wide confidence intervals, since bDMARD only was less commonly in use than an sDMARD or their combination. Eyes assessed under bDMARD (only) exposure were associated with a 50% to 25% lower risk of more severe SUN scores between months 3 and 30 of the observation compared to eyes assessed under no DMARD exposure. In later months, this risk tended to be comparable for the two conditions, but exposure to bDMARD was consistently associated with a lower rate of TCS use—the rate appeared 65–25% lower across the time points. Since the rate of TCS use was estimated while accounting for the concurrent SUN score, data are in line with the expectations based on the randomized trial of adalimumab vs. placebo: at a comparable level of AC inflammation, DMARD-exposed vs. non-exposed patients are likely to use relatively less TCS, or, if the TCS use is comparable, DMARD exposure vs. non-exposure is likely to be associated with less severe AC inflammation. The observed patterns reflect the daily-life development in which the disease status prompts (“causes”) use of topical or systemic treatments, which in turn reduces the disease intensity; hence, at certain time points, exposure (expected to be therapeutic) might transiently associate with poorer disease status, or appear to produce no benefit regarding either AC inflammation, or TCS use, or both, but over segments of time, the association of treatment with some form of benefit is apparent.

**Figure 2:**
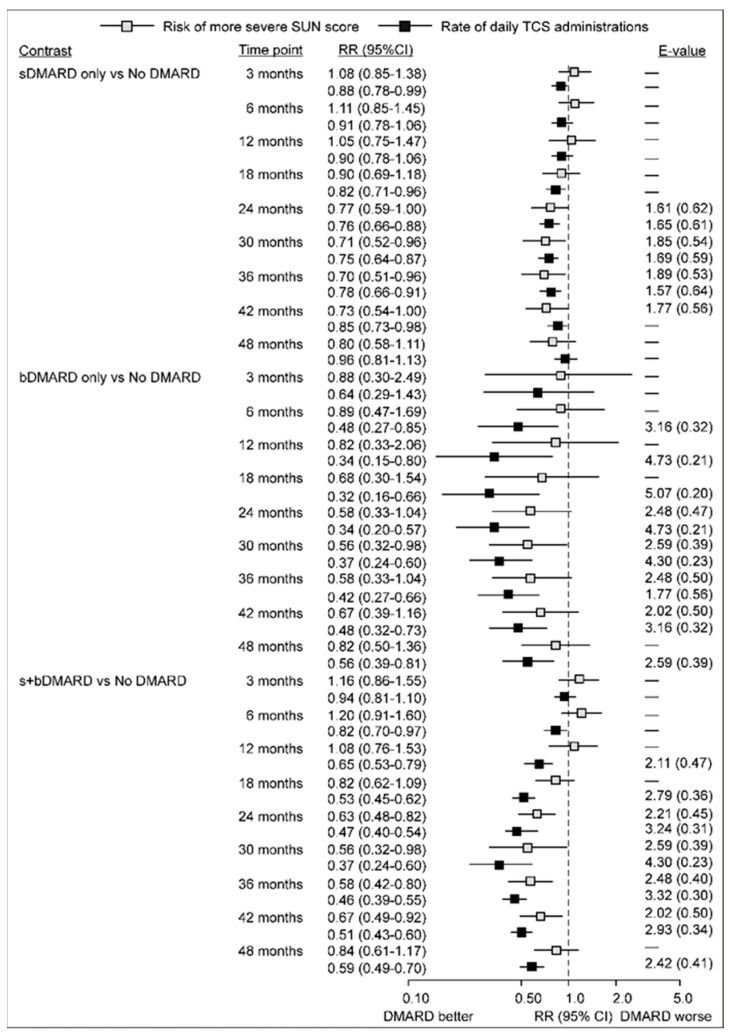
Adjusted association between exposure to DMARDs and the probability of having a SUN score > 0, and the number of daily topical corticosteroid (TCS) applications: differences between eyes assessed when sDMARD only, or bDMARD only, or both s+bDMARDs were in use, and eyes assessed when no DMARDs were in use. The two outcomes were analyzed jointly in a multivariate model for binary and count data. Estimates are relative risks and rate ratios (RR), respectively. E-values were calculated only for point-estimate RRs < 0.80 with upper CI limit < 1.0 or slightly >1.0, as a strength of association of residual confounder(s) needed to “push” the observed point-estimate to RR = 0.90 (considered a likely irrelevant difference). Estimates ≥ 0.80 (regardless of upper CI limit) were close to 0.90 anyway. The first number indicates association with the exposure (fold difference in prevalence of the confounder among the exposed compared to controls), and the bracketed number (its inverse) is the strength of the confounder effect (as RR). (s,b)DMARD—synthetic/biological disease-modifying antirheumatic drugs; SUN—standard uveitis nomenclature; TCS—topical corticosteroids.

**Figure A3:**
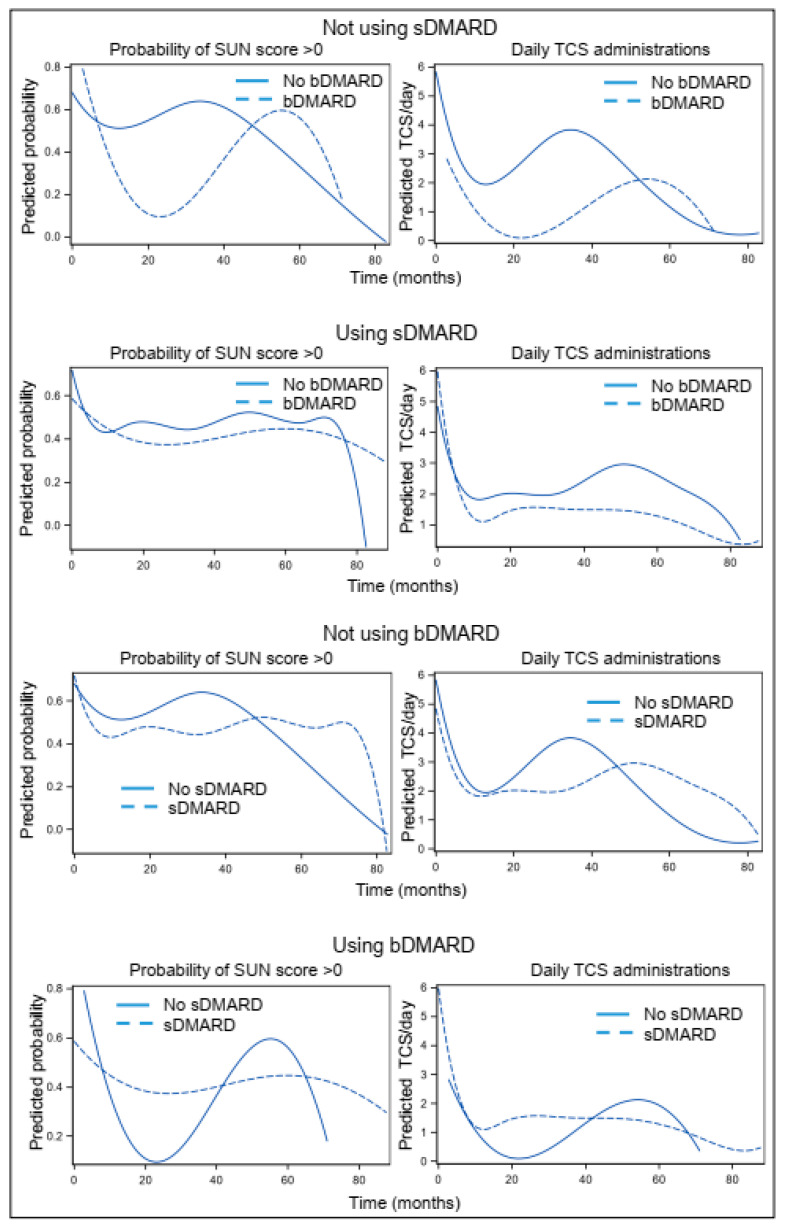
Adjusted predicted probabilities of AC SUN score > 0 and predicted number of daily TCS applications over time for eyes assessed while sDMARDs, bDMARDs, both, or none were in use: bDMARD vs. no DMARD without or with the use of sDMARD, and vice versa.

Figure 2 summarizes the estimated differences between sDMARD, bDMARD, or s+bDMARD exposure and no DMARD exposure at different time points assessed in a model that jointly analyzed the probability of having a SUN score > 0 and TCS daily applications (Appendix C, Figure A3 illustrates predictions across the entire observed period). Generally, the observed patterns are similar to those in Figure 1, but with some specifics. At the earlier time points, both outcomes appeared similar in eyes assessed under DMARD exposures and eyes assessed with no DMARD exposure. This might be due to the fact that 27/38 children had incident JIA-U, and 11 were referred to us from other institutions due to unsuccessful treatment; hence, the start of the present observations coincided with the start of adequate treatment. Differences between exposure to sDMARD alone and no DMARD were apparent at month 24 to month 42 of observation: the probability of SUN scores > 0 was 30% to 27% lower, and the rate of TCS daily applications was 15% to 25% lower with exposure to sDMARD (Figure 2). Differences between exposure to bDMARD alone and no DMARD in TCS use were apparent at month 6 to month 48: the rate of daily applications was lower by 52% to 68% (Figure 2). Differences in the probability of SUN scores > 0 were apparent at months 24, 30, and 36 (42–44% lower) (Figure 2). Differences between exposure to both sDMARD and bDMARD and no DMARD in TCS use were apparent at months 12 to 42: the rate of daily applications was lower by 35% to 63% (Figure 2). Differences in the probability of SUN scores > 0 were apparent at months 24, 30, 36, and 42 (33% to 42% lower) (Figure 2).

## 4. Discussion

In the present study we aimed to assess the benefits of systemic IMTs, i.e., sDMARDs and bDMARDs, that one could reasonably expect in the real-life treatment of children with JIA-U. We focused on two straightforwardly identifiable and practically meaningful outcomes commonly used to guide the use of topical or systemic treatment [31,33,52,53]: the intensity of AC inflammation (the SUN score) and intensity of TCS use represented by the total number of applications over 24 h. Treatments (exposures) of interest were defined in line with the up-to-date practice regarding IMT use in this indication [2,8,9,19]: sDMARDs (preferably MTX) are the first-line IMT choice; bDMARDs (preferably anti-TNF-alpha compounds) are an alternative (contraindications or adverse effects of sDMARD, inefficacy), or an add-on treatment. They were assessed in comparison to no exposure to DMARDs. Hence, we report on medically relevant exposures and outcomes, where neither was susceptible to misclassification.

Assessing treatment effects implies the estimation of causal effects, a goal difficult to achieve in non-randomized settings. Even under “ideal” circumstances, such efforts are susceptible to a number of biases. With this in mind, we interpret the present estimates as indicators of the strength of association between the exposures and the outcomes of interest. However, the biological and medical plausibility of actions undertaken in the management of children suffering JIA-U over time strengthens the view that these associations predominantly reflect treatment effects.

The present cohort included all children with JIA-U on systemic IMT managed at our institution over the index period, with only a few exceptions (when the observed period was shorter than 3 months, or AC inflammation was physically impossible to assess). Hence, they represent a sample of unselected consecutive children, most of whom were incident JIA-U patients (27/38). Over time, however, some form of patient selection most likely occurred: those achieving remission were likely less commonly seen or attended fewer/no ophthalmological visits, whereas those with more intense JIA-U might have been assessed more frequently and over a longer period of time. The apparent loss of association between DMARD exposure and lower probability of more severe SUN score (Figure 1) or score > 0 (Figure 2) supports such a view: the estimates at month 42 and 48 could have been based on data collected in children with difficult-to-treat AC inflammation who likely did not benefit much in this respect, although the association with less-frequent TCS use seemed clear. These observations are in agreement with the fluctuating nature of JIA and JIA-U [8], which are conditions that require flexible treatments (types and dosage). In this regard, the present data resemble a pragmatic trial with treatments guided by the development of the disease and treatment tolerability, with no restrictions imposed. Under such circumstances, associations between the exposures and outcomes at some time points might be due to reverse causation, while a lack of such associations (despite the expectations based on the current knowledge) might be due to the fact that the moment of assessment represents a “new baseline” as a consequence of a relapse of a previously (due to treatment) remitted disease. These facts may be viewed as limitations of the present study in the sense that they obscure quantification of causal treatment effects. On the other hand, they may also be considered as strengths, since they reflect developments of the disease in JIA-U patients over time. Finally, these characteristics distinguish the present study from most prior observational studies (also susceptible to various biases), which typically assessed outcomes at single time points, failing to capture clinically meaningful variation across variably timed prescheduled examinations—variation that may reflect transient spikes in inflammation, subsequent intensified corticosteroid exposure, and increased risk of ocular complications.

The present study is based on a sample of patients that might be considered reasonably large for a rare disease, with repeated ophthalmological assessments over a reasonably long period. As such, we believe it is more informative about treatment effectiveness (ability of a treatment to control the disease in daily life) than the observations focused on single time points [54].

Further strengths of the present study arise from the fact that we accounted for a number of time-varying covariates that could be reasonably viewed as confounders, and that the generated estimates showed closely similar patterns and values with two different data analysis approaches (i.e., were not model-dependent). When the estimates suggested associations between the exposure and outcomes, the E-values were rather high, indicating reasonable “resistance” of the estimates to residual confounding. Although this fact does not render the interpretation of the associations to be causal (in the treatment–outcome direction), it does support a conclusion that these associations were very likely “independent associations”.

Our present observations are in agreement with expectations based on the current knowledge and experience regarding the benefits of DMARDs in children with JIA-U [2,20,21,22,34,35]. Considering the lack of data, they cannot be assessed in relation to effects estimated in randomized trials, but under the elaborated conditions—since the rate of TCS use was estimated while accounting for the concurrent SUN score—the data are consistent with the expectations based on the randomized trial of adalimumab vs. placebo [22]: DMARD-exposed vs. non-exposed patients were likely to have less-severe AC inflammation and to use relatively less TCS. Therefore, our study enables certain reasonably supported conclusions: (i) over time, children with JIA-U treated with sDMARD (typically MTX, compared to no DMARD) should be expected to experience a relevant reduction in the need for TCS. This benefit may fluctuate due to a variety of reasons, but it could amount to as much as 25% reduction in TCS use. This may also be accompanied by a reduction in AC inflammation indicated by, e.g., an around 15% lower probability of “more severe” inflammation (e.g., SUN score 1 or higher vs. 0.5 or 0, or as SUN score 0 vs. any score > 0), or a maintenance of stable low-level AC inflammation. These expectations assume adequate sDMARD compliance, not compromised by disruptions; (ii) under the same conditions, children treated with bDMARD (typically anti-TNF-alpha compounds) should be expected to experience even greater average reductions in TCS use (e.g., up to a 50% or 60% reduction), with concomitant clinical improvements indicated by reduced probability (e.g., 30–40% on average) of more severe SUN scores, or any score > 0; (iii) children exposed to combined sDMARD and bDMARD could be reasonably expected to experience similar benefits regarding JIA-U.

To date, many other studies have suggested good effectiveness of IMT in controlling JIA-U but have reported short-term, methodologically heterogeneous observational results with likely considerable biases. As such, whether considered individually or jointly (e.g., in systematic reviews), they do not allow for uniform or subpopulation-specific estimates of treatment benefits of IMT (sDMARD, bDMARD) that one could consider to be most likely accurate [8,20,21,34,35,36,37,55,56]. Consequently, an urgent need for well-designed, long-term, and pragmatic comparative trials with standardized methodological features has been repeatedly advocated [8,34,35,55,56].

The only published RCT on the topic (evaluating AC inflammation and TCS use as outcome measures) [22] is a formal regulatory phase III trial of adalimumab vs. placebo in a moderately sized sample of children with JIA-U on “background” MTX treatment, with at least SUN grade 1+ AC inflammation, and a limited topical (and restricted during the trial) and systemic glucocorticoid use (intensification prohibited during the trial). Over an 18-month period, the cumulative risk of treatment failure (no improvement or worsening of inflammation by several criteria) was 60% lower in the treated children (RR = 0.40, 95% CI 0.22–0.73) [22]. At the same time, among children using TCS at a rate of at least one drop/day, reduction to zero drops/day was achieved in 47% of treated and 16% of control children (i.e., a three-fold higher probability) [22]. In the present study, periods “on bDMARD” or “on bDMARD + sDMARD” were compared to periods with no DMARD, which, to some extent, corresponds with the RCT: the peak benefits (although differently defined) in AC inflammation associated with the exposure were around 50% less inflammation, and the peak TCS use benefits were around a 60–65% reduction (this would correspond to a three-times-higher probability of reduced/none TCS use, e.g., 1/(1 − 0.65) = 2.9). In this respect, it seems plausible to conclude that the present observations are in agreement with the RCT results, not only qualitatively, but also quantitatively.

## 5. Conclusions

In this longitudinal observation study, we evaluated 38 children aged 1.3 to 15.2 years with 69 eyes affected with JIA-U for a total of 1970 eye examinations (minimum 6, maximum 59, median 16) irregularly scattered across 4.4–87.6 months (median 21.6 months) of follow-up. With adjustment for time-invariant (age at the start of observation, age at JIA onset, age at JIA-U onset, whether JIA was oligoarticular) and time-varying covariates (topical and systemic treatments, local ophthalmological conditions), periods of exposure to sDMARD (MTX) vs. no DMARD exposure were associated with peak benefits of 15–20% lower probability of having more severe AC inflammation (SUN score 1+ vs. 0.5 vs. 0), and a similar relative reduction in TCS daily applications. Periods of bDMARD exposure or of bDMARD+sDMARD exposure vs. no DMARD use were associated with peak benefits of around 50% reduction in probability of having more severe AC inflammation, and peak benefits of around 60–65% reduction in TCS use. The observations regarding bDMARD (only) or bDMARD + sDMARD exposure are in agreement with the extent of benefit suggested for adalimumab vs. placebo (+background MTX) in a randomized trial [22].

We underscore the need for harmonized standards in future JIA-U research. Unified protocols for database construction, outcome selection, and statistics methodology are essential to improve comparability and clinical interpretability across studies. We hope that the insights provided here—both regarding data structures and analytical approaches—will contribute to the ongoing development of consensus recommendations and enhance the quality of evidence in this field.

## Figures and Tables

**Table 1:** Characteristics of the included children and their eyes at the start of observation. Data are counts (percent), median (range).

Children						Eyes	
N	38		*Uveitis*			N	69
Girls	32 (84.2)		Incident, no complications	16 (42.1)		SUN score	1 (0–3)
Age at enrollment (yrs)	4.9 (1.3–15.2)		Incident, with complications	11 (28.9)		0	9 (13.0)
Age at JIA onset (yrs)	2.5 (1.1–12.6)		Prevalent, treated elsewhere	11 (28.9)		0.5	20 (29.0)
*JIA type/subtype*			Total with complications	19 (50.0)		1	26 (37.7)
Oligoarticular	21 (55.2)		*Systemic therapy*			2 (or 3 sporadically)	14 (20.3)
ANA negative	1		Any sDMARD	24 (63.1)		*Uveitis complications*	33 (47.8)
ANA positive	6		Methotrexate	23 (60.5)		Cataract	17 (26.4)
ANA & HLA B27 positive	1		Treatment duration (yrs)	2.0 (0–5.8)		Status post cataract op	5 (7.3)
Unknown subtype	13		Leflunomid	1 (2.6)		Synechiae	20 (29.0)
Polyarthritis overall	7 (18.4)		Mycophenolic acid	0		Keratopathy	15 (21.7)
ANA negative	3		Glucocorticoids	4 (10.5)		Glaucoma	(4.4)
ANA positive	1		NSAIDs	18 (47.4)		*Topical treatment*	
Unknown subtype	3		Any bDMARD	8 (21.0)		TCS drops	61 (88.4)
Spondylarthritis overall	2 (5.3)		Adalimumab	4		Daily applications	4 (0–10)
HLA B27 positive	1		Infliximab	0		TCS ointment	52 (75.4)
Unknown subtype	1		Etanercept	3		Daily applications	1 (0–10)
Systemic arthritis overall	2 (5.3)		Tocilizumab	1		Total TCS daily	6 (0–20)
Unknown JIA type	6 (15.8)		Use TCS to at least one eye	38 (100)		Mydriatic	50 (72.5)
Age at JIA-U onset (yrs)	4.4 (1.3–14.1)		Use TCS drops & ointment	33 (86.8)		Daily applications	3 (0–5)

ANA—anti-nuclear antibody; (s,b)DMARD—synthetic/biological disease-modifying antirheumatic drugs; HLA—human leukocyte antigen; JIA/JIA-U—juvenile idiopathic arthritis-associated uveitis.

**Table 2:** Pattern of data relevant for the present analysis over the observed time. Data are counts or median and range. When data were contributed by 3 or fewer children/eyes, individual values are listed.

	Time in Observation (Months)														
	To 6	6<-12	12<-18	18<-24	24<-30	30<-36	36<-42	42<-48	48<-54	54<-60	60<-66	66<-72	72<-78	78<-84	84<-90
Children	38	33	29	28	26	24	23	21	18	15	11	8	5	2	1
Total visits	244	132	93	92	96	83	65	67	56	36	37	14	11	6	3
Visits/child	6.5, 1–12	4, 1–10	2, 1–12	3, 1–8	2.5, 1–10	3, 1–10	3, 1–9	3, 1–8	2.5, 1–9	2, 1–10	3, 1–8	2, 1–3	1, 1–6	1 and 5	3
Eyes examined	476	263	180	174	160	159	123	129	105	70	74	26	21	7	3
SUN score															
0	197	136	95	83	98	84	73	57	61	34	39	18	13	4	0
0.5	177	106	64	72	48	61	36	53	37	29	26	7	8	3	3
1	81	20	18	19	18	14	13	15	7	5	7	1	0	0	0
2 or 3	21	1	3	0	0	0	1	4	0	2	2	0	0	0	0
TCS-treated eyes	382	191	125	119	109	103	64	87	76	54	52	10	16	3	2
TCS applic./day	3, 0–20	2, 0–11	1, 0–17	2, 0–10	1, 0–10	1, 0–8	1, 0–12	2, 0–24	2, 0–10	2, 0–12	1, 0–6	0, 0–4	1, 0–4	1, 0–4	0, 1 and 2
Mydriatic use	322	163	104	109	100	94	59	83	68	51	47	10	8	0	0
Top.NSAID	30	22	19	23	17	12	13	13	8	10	0	0	0	3	0
Keratitis	88	47	41	54	41	49	39	52	44	20	18	5	4	2	0
Cataract/post-	93	84	96	102	83	71	61	70	49	32	33	13	9	2	0
Synechiae	120	59	58	68	55	69	54	73	62	23	30	10	11	7	3
Glaucoma	42	47	49	60	33	51	32	37	34	14	21	9	17	6	3
Oligoarthritis	263	162	98	83	100	85	65	86	79	50	48	16	14	2	0
sDMARD in use	348	209	151	135	133	129	105	105	95	54	50	14	19	5	3
bDMARD in use	111	85	55	63	63	62	65	67	67	40	36	18	5	4	3
Steroids in use	88	19	9	14	14	14	8	12	33	18	10	4	8	0	0
NSAIDs in use	215	123	110	84	92	86	56	50	60	28	34	16	9	5	3

(s,b)DMARD—synthetic/biological disease-modifying antirheumatic drugs; TCS—topical corticosteroids; top.NSAID—topical nonsteroidal anti-inflammatory drug.

**Table 3:** Adjusted ^1^ predicted probabilities of AC SUN score categories and predicted average numbers of daily TCS applications at specific time points of observation (as time in months elapsed since the first visit during the index period).

	No DMARD	sDMARD	bDMARD	Both DMARDs
At 3 months				
SUN 0	0.327	0.485	0.662	0.638
SUN 0.5	0.547	0.446	0.303	0.324
SUN 1+	0.126	0.069	0.034	0.038
TCS/day	2.2	2.2	1.3	2.3
At 6 months				
SUN 0	0.351	0.496	0.666	0.642
SUN 0.5	0.534	0.438	0.300	0.320
SUN 1+	0.115	0.066	0.034	0.038
TCS/day	1.8	1.9	0.8	1.8
At 12 months				
SUN 0	0.400	0.520	0.672	0.650
SUN 0.5	0.505	0.419	0.295	0.314
SUN 1+	0.095	0.061	0.033	0.036
TCS/day	1.5	1.6	0.5	1.4
At 18 months				
SUN 0	0.452	0.543	0.678	0.657
SUN 0.5	0.470	0.401	0.290	0.308
SUN 1+	0.078	0.056	0.032	0.035
TCS/day	1.6	1.6	0.7	1.3
At 24 months				
SUN 0	0.504	0.565	0.684	0.665
SUN 0.5	0.432	0.384	0.285	0.301
SUN 1+	0.064	0.051	0.031	0.034
TCS/day	1.8	1.6	1.0	1.3
At 30 months				
SUN 0	0.557	0.588	0.690	0.672
SUN 0.5	0.390	0.365	0.280	0.295
SUN 1+	0.053	0.047	0.030	0.033
TCS/day	2.0	1.7	1.3	1.3
At 36 months				
SUN 0	0.608	0.610	0.696	0.680
SUN 0.5	0.349	0.347	0.274	0.288
SUN 1+	0.043	0.043	0.030	0.032
TCS/day	2.1	1.7	1.5	1.3
At 42 months				
SUN 0	0.657	0.632	0.702	0.687
SUN 0.5	0.308	0.329	0.269	0.282
SUN 1+	0.035	0.039	0.029	0.031
TCS/day	2.1	1.8	1.6	1.3
At 48 months				
SUN 0	0.703	0.653	0.708	0.694
SUN 0.5	0.268	0.311	0.264	0.276
SUN 1+	0.029	0.036	0.028	0.030
TCS/day	2.0	1.8	1.4	1.3

^1^ Covariates of all variables listed in Table 2, see Section 3.1. Data analysis for details (s,b)DMARD—synthetic/biological disease-modifying antirheumatic drugs; SUN—standard uveitis nomenclature; TCS—topical corticosteroids.

## Data Availability

The data underlying this article are available in the article. Further inquiries can be directed to the corresponding author.

## Abbreviations

The following abbreviations are used in this manuscript:

AC	Anterior chamber
bDMARD	Biologic disease-modifying antirheumatic drug
IMT	Immunomodulatory treatment
JIA	Juvenile Idiopathic Arthritis
JIA—U	Juvenile Idiopathic Arthritis-associated Uveitis
MTX	Methotrexate
NSAID	Nonsteroidal anti-inflammatory drugs
RCT	Randomized controlled trial
sDMARD	Conventional synthetic disease-modifying antirheumatic drug
SUN	Standard Uveitis Nomenclature
TCS	Topical corticosteroids

## Appendix A

**Table A1:** Selected data from pediatric observational studies of JIA-U patients receiving biologics showing diversity of methodology among studies.

Source	Study Type	Systemic Treatment	Collected Data	Frequency of Follow-Up Visits/Data Recording	Outcomes	Length of Follow-Up (Months)
Cecchin et al. 2018 [40]	Retrospective cohort study	ADA or IFX	AC SUN inflammation grade: YES, TCS daily dose: YES, Systemic treatment on each control: YES	At baseline and then once a week to once every 3 months, BUT data displayed cumulatively for the whole period	Number of uveitis flares, clinical remission, new onset or worsening of preexisting ocular complications, drug-related adverse events	24
Zannin et al. 2013 [41]	Prospective cohort study	ADA or IFX	AC SUN inflammation grade: YES, TCS daily dose: YES, Systemic treatment on each control: YES	At least every 3 months	Uveitis remission: the absence of active uveitis for more than 6 months on systemic treatment and without or on minimal topical treatment (corticosteroid and/or mydriatic-cycloplegic eyedrops less than once daily). The uveitis disease course; SUN Working Group criteria.	12
Tynjälä et al. 2008 [42]	Retrospective case series	ADA	AC SUN inflammation grade: AND Rao and Nussenblatt, TCS daily dose: NO	Every 4–12 weeks, but AC grade per patient separately at start, AT 12th and AT the end of follow-up	SUN improvement of uveitis activity; Change in uveitis flares	18.7 (4.5–35.6)
García-De-Vicuña et al. 2013. [43]	Prospective case series	ADA	AC SUN inflammation grade: YES, Mean immunosuppression load	Data compared on baseline and last visit	Mean degree of intraocular inflammation of the anterior chamber between the initial visit and at the end of follow-up	At least 6 months
Lerman et al. 2013 [38]	Retrospective cohort study, JIA-U and other pediatricnon-infectious uveitides	IFX, ADA, ETA +/− sDMARD +/− systemic steroids	AC SUN inflammation grade: YES, TCS daily dose: YES, Systemic treatment on each control: YES	Visit frequency varied, and, for survival analyses, data were used from clinical visits while a subject was on therapy until uveitis quiescence. All visits, but data categorized as inactive, slightly active, and active	Treatment success that sustained for at least 28 days	33.73 person-years
Gallagher et al., 2007 [44]	Retrospective case series	ADA, INF, daclizumab	AC Foster and Vitale inflammation grade, at each visit: YES, TCS daily dose: YES, All systemic treatment on each control: YES	Four to six-week intervals	Time to control inflammation; Improvement (at least 1+ change) in AC inflammation	16.9 months (range 1.3–54.1 months).

AC: anterior chamber; ADA—adalimumab; ETA—etanercept; IFX infliximab, JIA-U: juvenile idiopathic arthritis-associated uveitis; sDMARD—synthetic disease-modifying antirheumatic drug; TCS: topical corticosteroids.

## Appendix B

**Table A2:** Example from Dataset That Illustrates All Data for Selected Parameters in Two JIA-U Patients.

	**Patient No 1, Right Eye**					
**Examinations (No of Weeks from 1st Exam)**	**sDMARD**	**bDMARD**	**AC SUN Score, Right Eye**	**TCS Dose (Drops)**	**TCS Dose** **(Ointment)**	**Comment on the Disease Course**
1st exam	0	0	2	10	2	JIA-U detected on JIA-U screening, TCS introduced
3	0	0	1	5	2	
7	0	0	1	4	2	
11	0	0	0.5	3	1	
13	0	0	0.5	3	0	
14	0	0	0.5	2	0	
16	0	0	1	5	2	
18	1	0	1	4	2	Deterioration of JIA, no uveitis control, MTX introduced
20	1	0	1	4	2	
24	1	0	0.5	3	1	
23	1	0	0.5	2	1	
29	1	0	1	4	2	
35	1	0	1	5	2	
42	1	1	1	4	2	Deterioration of JIA, no uveitis control, ADA introduced
48	1	1	0.5	3	1	
55	1	1	0.5	2	0	
68	1	1	0.5	2	0	
77	1	1	0.5	2	0	
85	1	1	0	2	0	
97	0	0	0	1	0	Short-term break in sDMARD and bDMARD because of the flu
100	0	0	1	5	2	
101	1	1	0.5+	2	1	
102	1	1	0.5+	1	0	
104	1	1	0	1	0	
110	1	1	0	0	0	
121	0	1	0	0	0	Short-term break in MTX use (increased liver enzymes)
123	0	1	0.5+	2	1	
125	1	1	0.5+	2	0	
128	1	1	0	1	0	
135	1	1	0	0	0	
158	1	1	0	0	0	
164	1	1	0	0	0	
175	1	1	0	0	0	In general, compliance with keeping examination appointments was sometimes poor
	**Patient No 2, Right/Left Eye**					
**Examinations (No of Weeks from 1st Exam)**	**sDMARD**	**bDMARD**	**AC Grade**	**TCS Dose (Drops)**	**TCS Dose (Ointment)**	**Comment on the Disease Course**
1st exam	1	0	1/1	5/5	1/1	Patient was already followed due to JIA, receiving methotrexate, uveitis diagnosed
2	1	0	2/1	10/6	2/2	
3	1	0	0.5/0.5	5/3	1/1	
4	1	0	0.5/0.5	3/2	1/1	
6	1	0	0.5/0.5	2/1	0/0	
8	1	0	2/0.5	10/1	2/0	
9	1	0	1/0.5	6/1	2/0	
10	1	0	1/0.5	5/1	2/0	
11	1	0	1/0	5/1	2/0	
12	1	0	1/0	6/0	2/0	
13	1	0	0.5/0	4/0	2/0	
14	1	0	0.5/0	3/0	2/0	
16	1	0	0.5/0	3/0	1/0	
18	1	0	1/0.5	5/2	2/0	JIA in good control, but no uveitis control for the right eye on topical therapy + methotrexate
19	1	0	1/0	4/2	2/0	
21	1	0	0.5/0	4/1	1/0	
23	1	0	2/0	10/0	2/0	
24	1	0	1/0	8/0	2/0	No uveitis control, but patient has acute angina
25	1	0	1/0	6/0	2/0	
26	1	1	1/0.5	6/2	1/1	ADA introduced due to no uveitis control in right eye
27	1	1	1/0.5	5/2	1/0	
28	1	1	1/0.5	4/1	1/0	
30	1	1	0.5/0.5	3/1	1/0	Macular edema on right eye
32	1	1	0.5/0.5	2/1	1/0	
34	1	1	0.5/0	2/1	0/0	
37	1	1	0.5/0	1/0	0/0	
41	1	1	0/0	0/0	0/0	
44	1	1	0/0	0/0	0/0	
48	1	1	0/0	0/0	0/0	
51	1	1	0.5/0	3/0	0/0	
52	1	1	0.5/0	2/0	0/0	
54	1	0	1/0	5/0	1/0	Short-term break in both IMT because of the severe gastroenteritis
55	1	0	1/0	4/0	1/0	
56	1	1	0.5/0	2/0	1/0	
58	1	1	0/0	1/0	0/0	
61	1	1	0/0	0/0	0/0	
65	1	1	0/0	0/0	0/0	
69	1	1	0/0	0/0	0/0	Tocilizumab instead of ADA due to JIA deterioration and persistent macular edema
73	1	1	0/0	0/0	0/0	
77	1	1	0/0	0/0	0/0	
85	1	1	0/0	0/0	0/0	Macular edema resolution
93	1	1	0/0	0/0	0/0	
101	1	1	0/0	0/0	0/0	

AC: anterior chamber; ADA—adalimumab; bDMARD: biologic disease-modifying antirheumatic drug; JIA-U: juvenile idiopathis, arthritis-associated uveitis; sDMARD—synthetic disease-modifying antirheumatic drug; TCS: topical corticosteroids; 0—off treatment, 1—on treatment.

## Appendix C

Figure A1 and Figure A2 illustrate estimates over the entire observed period generated in a cumulative logit model fitted to AC SUN score data, and Poission model fitted to data on the number of daily (per 24 h) TCS applications, respectively. Figure A3 illustrates estimates over the entire observed period generated in a multivariate model for joint analysis of the probability of AC SUN score > 0 (binary) and number of daily TCS applications (Poisson). For the Poisson model, we considered that Poisson Chi^2^/DF = 0.90 indicated no overdispersion. To verify the proportional odds assumption, we fitted a generalized logit model with AC SUN score = 0.5 as the reference. We generated odds ratios indicating an increase in odds of scores of 0 vs. 0.5, and a reduction in odds of scores of 1 in favour of scores of 0.5 at time points in which the exposure–outcome association appeared obvious. As an example, the odds ratios indicating an increase in the odds of 0 and the inverse of the odds ratio representing a reduction in the odds of 1 at 24 months were similar, suggesting that the “exposure effect” in shifting probabilities from 0.5 to 0, and from 1 to 0.5 was similar (proportional odds assumption):

sDMARD + bDMARD vs. no DMARD: 1.68 and 1/0.555 = 1.80

sDMARD only vs. no DMARD: 1.14 and 1/0.74 = 1.35

bDMARD only vs. no DMARD: 2.41 and 1/0.43 = 2.33
